# Supplementary material for: Volcanism and long-term seismicity controlled by plume-induced plate thinning
Source: Nat Commun. 2025 Aug 22;16:7837. doi: 10.1038/s41467-025-62967-5 (PMC12373950; doi:10.1038/s41467-025-62967-5)
Supplement: Supplementary file 1 — Supplementary Information [file 41467_2025_62967_MOESM1_ESM.pdf]

Supplementary information for “Volcanism and long-term seismicity controlled by plume-induced plate thinning”

Raffaele Bonadio[1*], Sergei Lebedev[1,2], David Chew[3], Yihe Xu[4,1], Javier Fullea[5,2], Thomas Meier[6]

[1]Department of Earth Sciences, Bullard Laboratories, University of Cambridge, Madingley Road, Cambridge, CB30EZ, United Kingdom.
[2]School of Cosmic Physics, Dublin Institute for Advanced Studies, 5 Merrion Square North, Dublin, Dublin 2, Ireland.
[3]Department of Geology, Museum Building, Trinity College Dublin, Dublin, Dublin 2, Ireland.
[4]School of Earth Sciences, Yunnan University, Kunming, 650500, China.
[5]Department of Earth Sciences and Astrophysics, Universidad Complutense Madrid, Pl. de las Ciencias, 1, Moncloa, Aravaca, Madrid, 28040, Spain.
[6]Institute of Geosciences, Christian Albrecht University, Otto-Hahn Platz 1, Kiel, 24118, Germany.

*Corresponding author(s). E-mail(s): rb2075@cam.ac.uk;
Contributing authors: sl2072@cam.ac.uk; chewd@tcd.ie; xuyihe216@ynu.edu.cn; jfullea@ucm.es; thomas.meier@ifg.uni-kiel.de;

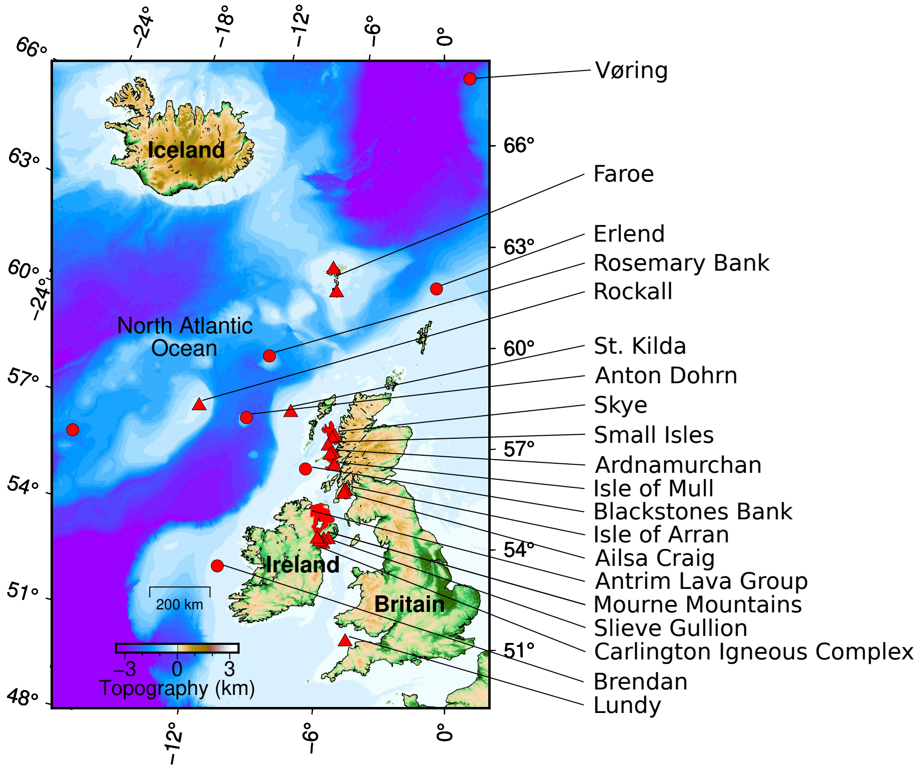

Supplementary Fig. 1 Topographic map (IOC, IHO, BODC, 2003. Centenary Edition of the GEBCO Digital Atlas, https://www.gebco.net, last accessed July 2025) of the Britain and Ireland area, with major NAIP volcanism locations identified. Major intrusions and submarine complexes are plotted with triangles and circles, respectively.

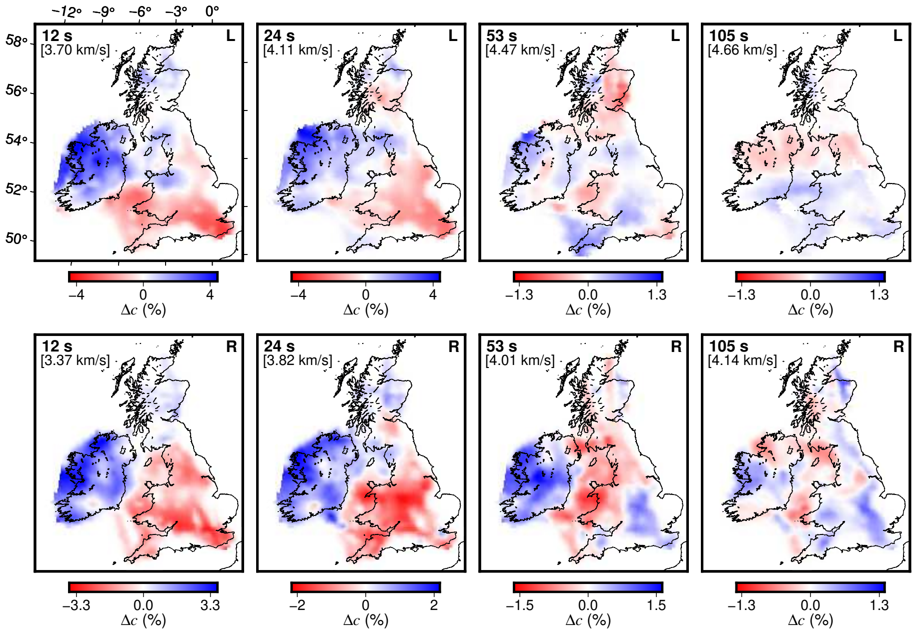

Supplementary Fig. 2 Phase-velocity maps of Love (top row) and Rayleigh (bottom row) surface waves at 4 example periods. The maps are calculated using the optimal resolution method that solves the Backus-Gilbert problem at every point, using directly estimated model errors and finding the phase-velocity value and the optimal averaging length at each point such that the model error is below a threshold [42, 43]. Each map's period and phase-velocity reference are specified in the top left corner of the frame.

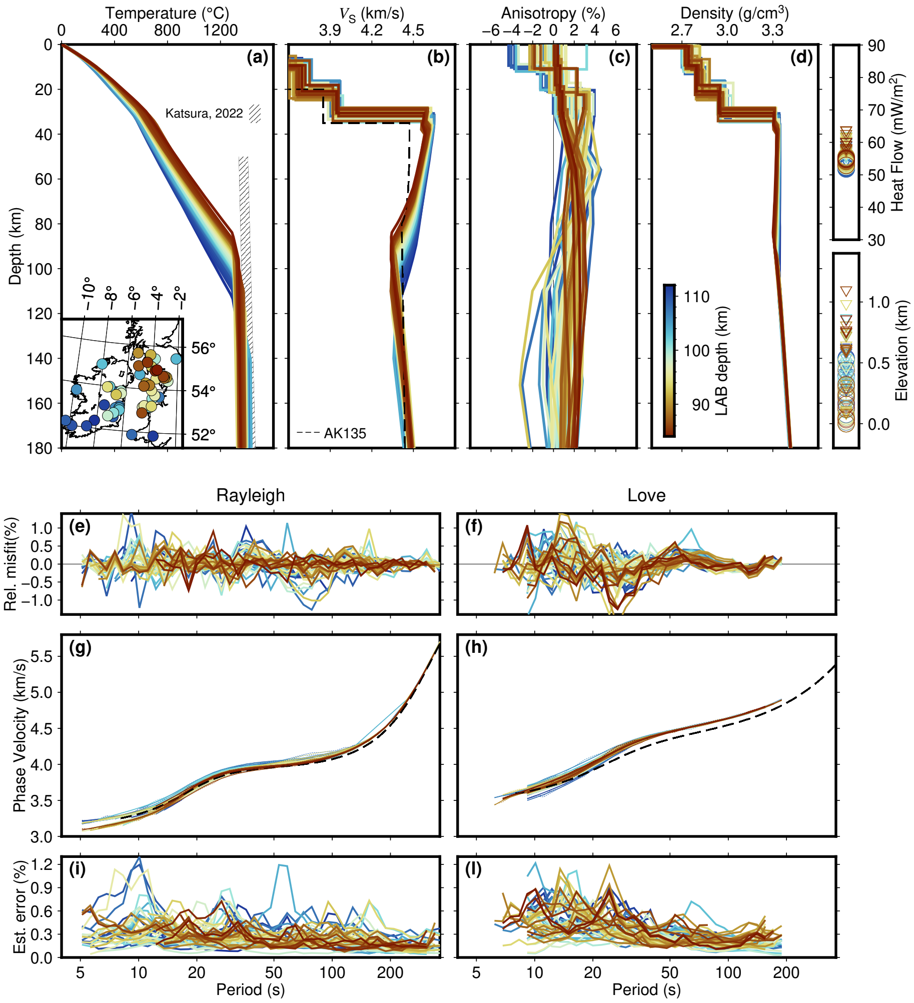

Supplementary Fig. 3 Thermodynamic inversions at the 53 locations where exhumation measurements are available (Fig. 2). The colour scale indicates the LAB depth, and the profiles are plotted in the order from the thick-lithosphere ones first to the thin-lithosphere ones last. (a-d) the profiles of temperature, S-wave velocity, radial anisotropy and density and the modelled (inverted triangles) and observed (circles) heat flow and surface elevation. (e-f) the synthetic-data misfits for Rayleigh and Love phase-velocity curves. (g-h) Rayleigh and Love phase-velocity curves, including data (solid lines) and synthetic (dashed). The misfits are typically within the line thickness and can be seen more clearly in the misfit plots above. (i-j) estimated, frequency-dependent errors of the Rayleigh and Love phase-velocity curves.

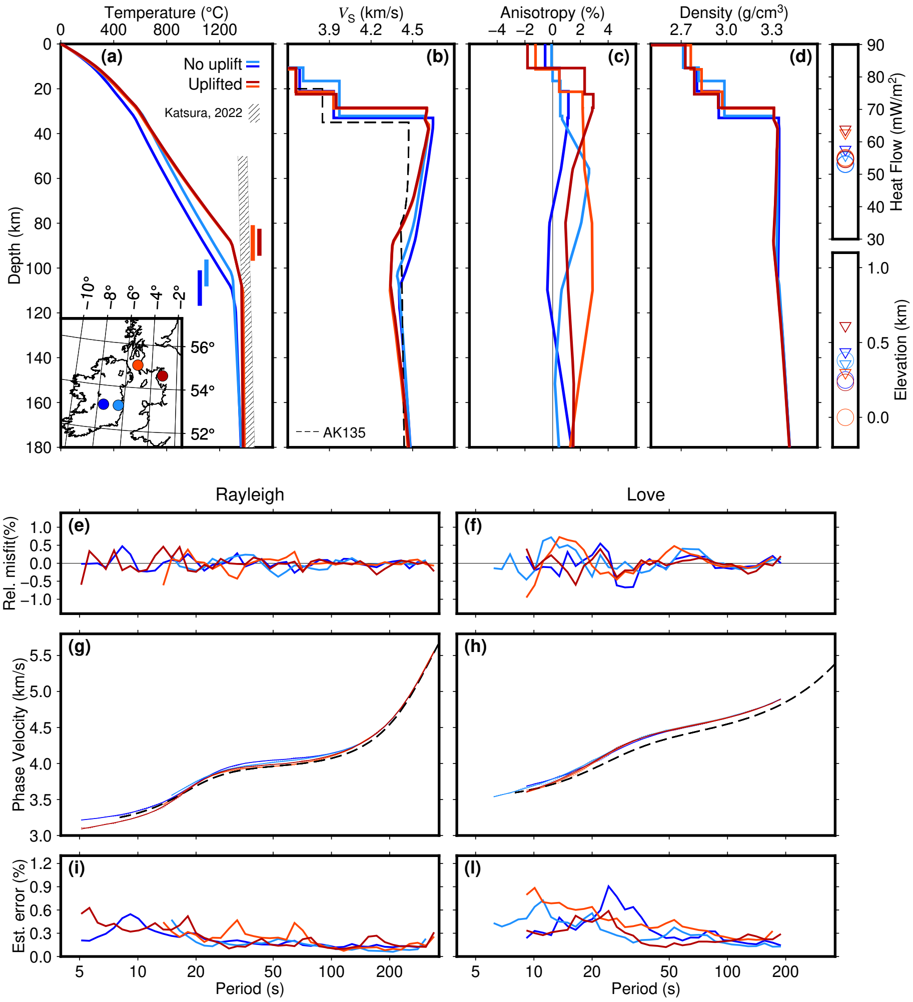

Supplementary Fig. 4 The full models given by the thermodynamic inversions at 4 example locations, summarised in Fig. 3. Two locations are where Paleocene uplift and magmatism took place (red) and two—where they did not (blue). (a-d) the profiles of temperature, S-wave velocity, radial anisotropy and density and the modelled (inverted triangles) and observed (circles) heat flow and surface elevation. Uncertainties on the lithospheric thickness are shown with the vertical bars. (e-f) the synthetic-data misfits for Rayleigh and Love phase-velocity curves. (g-h) Rayleigh and Love phase-velocity curves, including data (solid lines) and synthetic (dashed). The misfits are typically within the line thickness and can be seen more clearly in the misfit plots above. (i-j) estimated, frequency-dependent errors of the Rayleigh and Love phase-velocity curves.

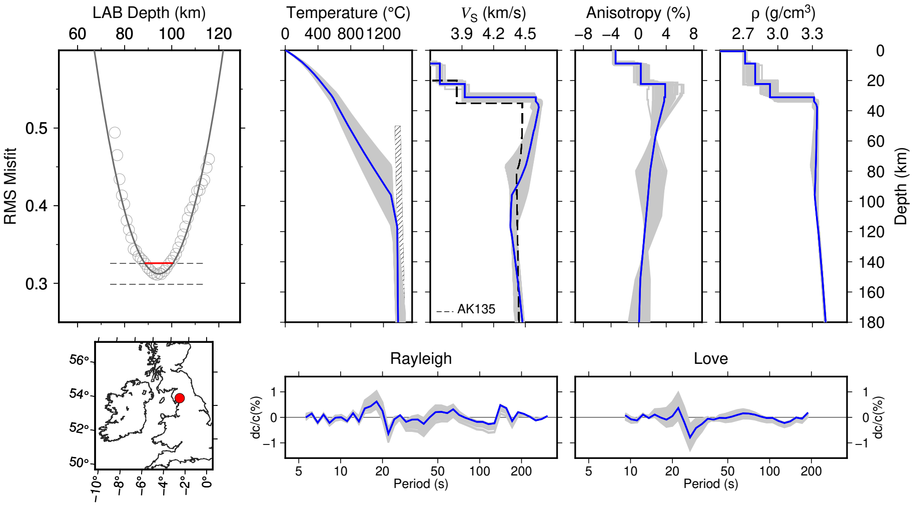

Supplementary Fig. 5 Estimation of the LAB depth uncertainty for a grid point in Britain. Blue lines: best-fitting, preferred model. Grey: the models yielded by inversions with the LAB depth fixed at values within a range around the best-fitting value. Red line: the uncertainty estimate, derived from the width of the RMS misfit parabolic curve.
